# Effects of Transcranial Direct Current Stimulation on Episodic Memory Related to Emotional Visual Stimuli

**DOI:** 10.1371/journal.pone.0010623

**Published:** 2010-05-13

**Authors:** Barbara Penolazzi, Alberto Di Domenico, Daniele Marzoli, Nicola Mammarella, Beth Fairfield, Raffaella Franciotti, Alfredo Brancucci, Luca Tommasi

**Affiliations:** 1 Department of Psychology, University “Alma Mater Studiorum” of Bologna, Bologna, Italy; 2 Department of Biomedical Sciences, University “G. d'Annunzio” of Chieti, Chieti, Italy; 3 Department of Neuroscience and Imaging, University “G. d'Annunzio” of Chieti, Chieti, Italy; University of Groningen, Netherlands

## Abstract

The present study investigated emotional memory following bilateral transcranial electrical stimulation (direct current of 1 mA, for 20 minutes) over fronto-temporal cortical areas of healthy participants during the encoding of images that differed in affective arousal and valence. The main result was a significant interaction between the side of anodal stimulation and image emotional valence. Specifically, right anodal/left cathodal stimulation selectively facilitated the recall of pleasant images with respect to both unpleasant and neutral images whereas left anodal/right cathodal stimulation selectively facilitated the recall of unpleasant images with respect to both pleasant and neutral images. From a theoretical perspective, this double dissociation between the side of anodal stimulation and the advantage in the memory performance for a specific type of stimulus depending on its pleasantness supported the specific-valence hypothesis of emotional processes, which assumes a specialization of the right hemisphere in processing unpleasant stimuli and a specialization of the left hemisphere in processing pleasant stimuli. From a methodological point of view, first we found tDCS effects strictly dependent on the stimulus category, and second a pattern of results in line with an interfering and inhibitory account of anodal stimulation on memory performance. These findings need to be carefully considered in applied contexts, such as the rehabilitation of altered emotional processing or eye-witness memory, and deserve to be further investigated in order to understand their underlying mechanisms of action.

## Introduction

In the present study we examined the effects of Transcranial Direct Current Stimulation (tDCS) on emotional memory. The tDCS is a non-invasive technique of brain stimulation recently reintroduced in neurophysiological research in virtue of the promising advantages it offers for both the rehabilitation of many diseases, and the study of cognitive processes, their neural substrates and related plasticity phenomena [1]–[3]. Stimulation was applied during the encoding of emotional pictures that differed for two affective dimensions: *arousal*, corresponding to the stimulus-induced psychophysiological activation (ranging from the calm of neutral stimuli to the high excitement of emotional ones), and *valence*, related to the degree of stimulus pleasantness (ranging from pleasant stimuli to unpleasant ones, with neutral stimuli in an intermediate position).

Besides the well-proved work in tandem of amygdala and hippocampus for the processing of emotional stimuli, including the formation of emotional episodic memories [4], [5], much evidence has supported a pivotal role of the prefrontal cortex in emotional stimulus evaluation [6], [7], an area also involved in the encoding phase of episodic memories (regardless of their emotional content) [8]–[11]. However, the specific contribution of each cerebral hemisphere in emotional stimuli processing continues to be controversial, and two main hypotheses have been proposed concerning the involvement of the left and right prefrontal regions. On one hand, much evidence supports the right-hemisphere hypothesis, which assumes that the right hemisphere is specialized in processing all emotional stimuli, independently of their pleasantness [12]. In line with this, the right hemisphere would be more sensitive to affective arousal (which distinguishes between emotional and non-emotional stimuli), than to affective valence. On the other hand, a number of convincing data suggest a valence-specific organization of emotional perception, with the left hemisphere specialized in processing pleasant and positive emotions and the right hemisphere specialized in unpleasant and negative ones [13]–[15].

To the best of our knowledge, tDCS studies on emotional memory are still missing, despite the high relevance of this topic, from both a theoretical and a more applied point of view. Although the precise action mechanisms of the tDCS are still not completely clear, the induction of a relatively weak constant current flow through the cortex, via scalp electrodes positively and negatively charged (i.e., anode and cathode, respectively), is supposed to reversibly modulate the underlying regional brain activity by modifying spontaneous neuronal excitability [16]–[20]. So far, most tDCS studies have investigated motor functions, and have obtained quite reliable results (i.e., facilitation of the contrololateral effector with respect to the side of the motor cortex exposed to anodal stimulation [21], [22]), but the effects of the mentioned tDCS parameters on other cognitive functions have not received comparable attention yet.

The investigation of memory with tDCS is only at the beginning and the panorama is complicated by the high number of systems and processes involved in memory architecture and by the complexity of the neuronal networks involved. In particular, except for a few studies that investigated more long-term memory systems [23], [24], most tDCS research in this field has focused on working memory processes and have generally reported that anodal stimulation of dorsolateral prefrontal cortex (a key region for temporary storage and manipulation of stimuli) improves behavioral performance in a wide range of tasks engaging working memory, both in healthy people [25], [26], and in different populations of patients [27], [28]. Nevertheless, data are not completely univocal and some studies have found contrary effects showing that both anodal and cathodal stimulations can interfere with working memory processes, thus impairing task execution [29], [31]. Therefore, a direct and univocal correspondence between anodal stimulation and beneficial/facilitatory effects and between cathodal stimulation and detrimental/interfering effects is far from being considered unquestionable. In addition to the polarity of the stimulation, effects of tDCS often depend on various factors such as current density, stimulation duration, orientation of the electric field, type of electrode montage, site of application, type of experimental task, and neural mechanisms under investigation.

As anticipated, the present research was aimed at investigating emotional memory through tDCS, by measuring the delayed free recall of affective stimuli with a twofold purpose. First, we aimed at verifying whether different stimulations of the two hemispheres could induce specific differences in the stimulus categories recalled, with reference to their affective arousal and valence, thus investigating the right-hemisphere hypothesis and/or the valence-specific hypothesis of emotional processing. With respect to emotional arousal, we expected that if anodal stimulation is effective in facilitating cognitive functions related to the stimulated area (as highlighted in many previous tDCS studies [16]–[19], [26]–[28]), then we should observe a retrieval improvement for emotional in comparison with neutral pictures following anodal stimulation of the critical right areas. With regards to emotional valence, we hypothesized that if anodal stimulation is effective in improving free recall, and if the two hemispheres are specialized in processing stimuli with opposite emotional valence (i.e. pleasant stimuli in the left hemisphere and unpleasant stimuli in the right hemisphere), then anodal stimulation of the critical right areas should selectively enhance retrieval of negative images, and anodal stimulation of the homologue left areas should selectively enhance retrieval of positive images. Second, we aimed at analyzing the effects of the stimulation on the formation of episodic memory in an explicit learning task. Indeed, due to the lack of standard tDCS protocols able to induce predictable effects, a systematic investigation of its parameters is needed in order to identify those more appropriate to influence each specific cognitive or affective process, both in healthy people and in patients with neurological or psychiatric diseases.

## Methods

### Participants

Twelve healthy participants (6 females, mean age: 26.83, SD: ±4.86, all right-handed) took part in the experimental research after giving their written informed consent in accordance with the principles of the Declaration of Helsinki and following the approval of the ethical committee. None of the individuals, naïve as to the purpose of the study, reported any history of neurological or psychiatric disease and of implanted metal objects.

### Transcranial direct current stimulation protocol

Transcranial direct current was delivered through a battery-driven constant current stimulator (DC-Stimulator, NeuroConn GmbH, Germany; distributed by EMS, Italy), using a pair of surface saline-soaked sponge electrodes (5 cm×7 cm). Following tDCS safety guidelines [3], a constant current of 1 mA (corresponding to a current density of 0.029 mA/cm^2^) was applied for 20 minutes in each experimental session (including 1 minute at the beginning and 1 minute at the end of treatment in which current was ramped up and down, respectively). With regards to electrode montage, we used bilateral, or dual-hemisphere, stimulation (i.e., anode and cathode placed over homologue areas of the two cerebral hemispheres), positioning the longest side of the electrodes horizontally between F3/4 and C3/4 sites of the International 10–20 System for EEG electrode placement. Given the great extension of the neuronal networks involved in both emotional stimulus evaluation and processing (i.e., frontal associative areas), and encoding and retention mechanisms related to episodic memories (i.e., dorsolateral prefrontal cortex and anterior temporal areas), the choice of our electrode position aimed at stimulating a cortical area which could comprise frontal and temporal regions at the same time. In addition, since we were interested in the specific role of each hemisphere not only with respect to arousal (emotional vs. neutral), but also to valence (pleasant vs. unpleasant), we stimulated both the right and the left areas. There are not many data available on optimal electrode arrangement in modulating non-motor functions (and in particular emotional memory) with tDCS, so we chose a bilateral stimulation, rather than a unilateral one, for two reasons. First, the bilateral montage allows us to control the investigated variables better, since it allows us to obtain a stimulation of equal spreading, and thus of equal intensity (although in the opposite direction), on the two hemispheres. On the contrary, unilateral montages (with one electrode on the target brain region and the other on a region, sometime erroneously, assumed not to be involved in the investigated processes) could give rise to uncontrolled effects simply linked to the inadequate positioning of the reference electrode. Second, we followed the more recent studies that have more systematically investigated the primary motor cortex using different tDCS protocols. Since the studies that achieved the more effective modulations on task execution were those that made use of one electrode placed over the primary motor cortex and the other placed contralaterally (i.e., addictive effects with respect to uni-hemispheric conditions) [32], we chose the same dual-hemisphere montage.

Our tDCS protocol included 3 experimental sessions, each corresponding to a stimulation condition, Administration order was counterbalanced across participants: (1) Anodal stimulation of right area between F4 and C4 and cathodal stimulation of left area between F3 and C3 (hereafter referred as “RA/LC” stimulation: Right Anodal/Left Cathodal stimulation); (2) Anodal stimulation of left area between F3 and C3 and Cathodal stimulation of right area between F4 and C4 (hereafter referred as “LA/RC”: Left Anodal/Right Cathodal stimulation) (3) Sham stimulation (hereafter referred as “S” stimulation), with electrodes placed in the same positions of the real stimulations (i.e., RA/LC arrangement for half participants and LA/RC arrangement for the other half). In this placebo condition, a stimulation of 1 mA was delivered for 30 seconds, which has been demonstrated to be unable to modulate cognitive functions, but is perceivable enough to give participants the impression of being stimulated [33]. Stimulation sessions were conducted, on three consecutive days, such that sessions of each participant were separated by at least 24 hours. Given that a biological variability in the circadian rhythm can influences cognitive functions, each participant underwent the stimulation at approximately the same hour in each of the three daily sessions.

**Figure 1:**
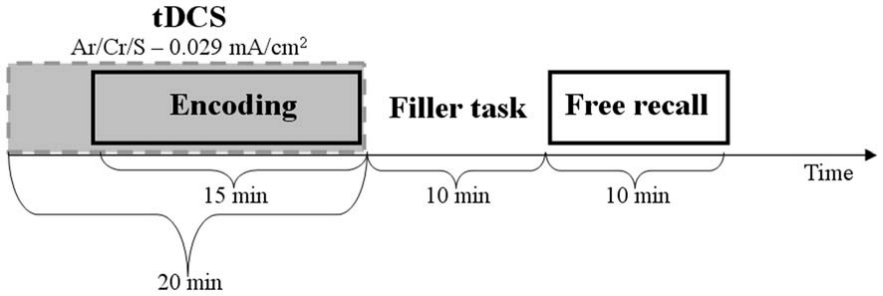
Experimental paradigm. The different phases of the experimental paradigm are reported along the time-line. Grey box corresponds to the time of tDCS stimulation, which included the encoding task (A: anodal stimulation; C: cathodal stimulation; S: sham stimulation).

As can be seen in Figure 1, five minutes after the beginning of the stimulation condition, the encoding phase (in which images with different emotional arousal and valence were displayed to participants) initiated as well. Picture encoding lasted for 15 minutes and finished synchronically with the stimulation. A visuo-motor filler task that lasted approximately 10 minutes, followed the stimulation, and finally participants performed the free recall test for a maximum of 10 minutes.

### Stimuli, task and procedure

Stimuli consisted of a sub-set of 96 images (24 pleasant, 24 unpleasant, and 48 neutral) selected from the International Affective Pictures System (IAPS) [34]. The images were chosen so that pleasant and unpleasant pictures differed from neutral pictures in terms of both emotional arousal and emotional valence, whereas pleasant and unpleasant pictures differed from each other only in term of emotional valence, but not of emotional arousal. The 96 pictures were divided into 3 lists of 32 stimuli (8 pleasant, 8 unpleasant, and 16 neutral), each presented in one of the three experimental sessions. To avoid primacy and recency effects, two filler images (not included in the recall analyses) preceded and followed the experimental pictures in every session. The images were sequentially displayed in a random order for 25 seconds each, without an inter-stimulus interval.

Participants were instructed to remember them by paying attention to both the main subject and the details for a subsequent delayed free recall test (intentional learning). This encoding phase (during which tDCS was applied, see figure 1) was followed by a visuo-motor filler task that lasted approximately 10 minutes (key pressing in response to the visual presentation of circles). The filler task was included only to avoid active memory strategies during the retention interval, which separated the encoding from the retrieval phases, therefore its analysis will not be reported since it is not designed to test our hypothesis about emotional memory. At the end of the filler task, participants were asked to remember as many pictures as possible during a maximum time interval of 10 minutes. Specifically, they were asked to write down every picture they could retrieve (without following presentation order), by describing the image details necessary, for an hypothetic outsider, to univocally identify it in the entire subset of the used pictures (which also comprises quite similar images). Two raters independently judged recall responses (by assigning one point for each correctly recalled picture), with a third rater being used in the event of a disagreement. Only pictures whose description was sufficiently detailed to allow their univocal identification were classified as remembered (i.e., when participants did not report the details necessary to distinguish an image from a similar one included in the stimulus set, that image was excluded from the calculation of the pictures correctly recalled).

### Data analyses

Statistical analyses were performed by using the percentages of pictures correctly recalled by participants as a function of both stimulation condition, and image category determined with respect to emotional arousal and emotional valence dimensions as the dependent variable. In order to test the effect of tDCS on emotional arousal, we collapsed pleasant and unpleasant images into a single category of emotional images (i.e., characterized by high arousal), and compared them to non-emotional, or neutral, images (i.e., characterized by low arousal). Therefore, a within-group Analysis of Variance (ANOVA) was carried out with the following two factors: Condition of Stimulation (3 levels: RA/LC vs. LA/RC vs. S) and Category of Image (2 levels: emotional images vs. neutral images). Differently, in order to test the effect of tDCS on emotional valence, we kept the emotional images with different pleasantness separate, and performed an ANOVA with Condition of Stimulation (3 levels: RA/LC vs. LA/RC vs. S) and Category of Image (3 levels: pleasant images vs. unpleasant images vs. neutral images) as within-group factors. The Huynh–Feldt correction was applied when sphericity assumptions were violated, and in these cases, the uncorrected degrees of freedom, epsilon values and the corrected probability levels were reported. Duncan's post-hoc comparisons were computed for significant ANOVA results.

## Results

The first ANOVA, testing possible differences in recall percentages depending on image emotional arousal, revealed a significant main effect of the factor Category of Image (F(1,11) = 17.32, p = 0.0016), emotional pictures being remembered better than non-emotional ones (61.63% and 51.04%, respectively), thus corroborating the solid effect of emotional arousal in enhancing memory performance. Instead, neither the effect of tDCS (F(2,22) = 2.82, p = 0.081), nor the double interaction Condition of Stimulation by Category of Image were significant (F(2,22) = 0.085, p = 0.92).

**Figure 2:**
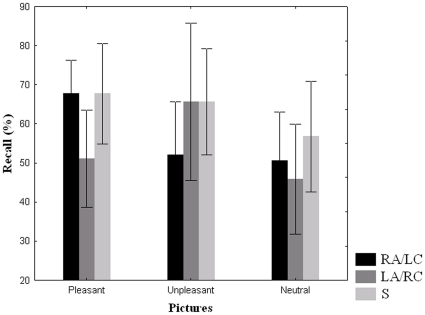
Task performance. Means and standard errors of retrieval percentages as a function of picture valence and stimulation conditions (RA/LC: Right Anodal/Left Cathodal stimulation; LA/RC: Left Anodal/Right Cathodal stimulation; S: sham stimulation).

The ANOVA which tested possible differences in recall percentages as a function of image emotional valence showed that the main effect of Condition of Stimulation did not produce significant differences on recall percentages (F(2,22) = 2.24, p = 0.13). On the contrary, the factor Category of Image reached significance (F(2,22) = 5.91, ε = 0.82, p = 0.014), neutral pictures being recalled significantly less (51,04%) than both pleasant and unpleasant pictures (62.15%, p = 0.0068 and 61.11%, p = 0.01, respectively), a result which further confirms the well-proved advantage of emotional over non-emotional stimuli in memory tests. More interestingly, the double interaction Condition of Stimulation x Category of Image was also significant (F(4,44) = 2.87, ε = 1, p = 0.034, see figure 2). In particular, post-hoc tests revealed that in the S condition there were no differences as a function of picture category whereas, on the contrary, the two real conditions of stimulation showed a similar pattern of results with regards to emotional memories. Specifically, in the RA/LC stimulation condition, pleasant images were remembered significantly better (67.71%) than both unpleasant and neutral images (52.08%, p = 0.032, and 50.52%, p = 0.022, respectively); whereas in LA/RC stimulation condition unpleasant images were remembered significantly better (65.62%) than both pleasant and neutral images (51.04%, p = 0.04, and 45.83%, p = 0.007, respectively). Turning the focus to the differences among stimulation conditions within each category of images, whereas neutral stimuli were not affected by the three different kinds of stimulation, pleasant images were recalled worse in the LA/RC stimulation condition (51.04%) than in the RA/LC and in the S conditions (p = 0.024 and p = 0.026, respectively), the last two conditions showing overlapped recall percentages (67.71%). The reversed pattern characterized unpleasant images, which were recalled worse in the RA/LC stimulation condition (52.08%) than in the LA/RC and in the S conditions (p = 0.049 and p = 0.057, respectively), the last two conditions exhibiting similar task performances (65.62%).

## Discussion

In the present tDCS study, healthy volunteers received fronto-temporal stimulation of both cerebral hemispheres during the encoding of pictures with different affective arousal and valence, in order to measure its effects on a following free recall task. Our first aim, more theoretically oriented, was to use brain stimulation to clarify the roles of the two hemispheres in the evaluation and processing of emotional stimuli, a controversial issue, with many data converging towards two theories only partially in opposition: the right-hemisphere hypothesis and the valence-specific hypothesis [35]. The analyses we performed (considering pleasant and unpleasant pictures separately and collapsing them into an emotional picture category) revealed that emotional stimuli, regardless of their valence, tend to be remembered better than non-emotional stimuli. This result confirms the well-proven advantage of emotional arousal in improving memory performances [36]. We hypothesized that if anodal stimulation facilitates episodic memory encoding of emotional images, then we should find better recall for emotional in comparison with neutral pictures during anodal stimulation of the critical right areas. However, as evidenced by the non-significant interaction between the kind of stimulation and the category of images (i.e., high vs. low arousal), the anodal stimulation of the right fronto-central regions did not selectively increase emotional picture recall. Therefore, our data does not seem to support the right-hemisphere hypothesis, which considers the right anterior cortical areas as fundamental in emotional arousal processing [12]. On the contrary, we found evidence in favor of the valence-specific hypothesis [13]–[15]. In line with this, and assuming beneficial effects of anodal stimulation on the following recall, we expected that anodal stimulation of the right areas would selectively enhance negative image retrieval, and anodal stimulation of the homologue left areas would selectively enhance positive image retrieval. The significant interaction between side of anodal stimulation and image category (found by analyzing pleasant and unpleasant pictures separately) confirmed that the two hemispheres have specific roles in processing stimuli with different valence, although the anodal stimulation effects seemed to be interfering, rather than facilitatory. We found a double dissociation between the stimulation of a specific hemisphere and the type (pleasant or unpleasant) of picture remembered better after each of the differently lateralized stimulations. In particular, right anodal/left cathodal stimulation of the fronto-central regions during emotional memory encoding selectively facilitated the recall of pleasant images with respect to both unpleasant and neutral images, whereas left anodal/right cathodal stimulation of the same areas selectively facilitated the retrieval of unpleasant images with respect to both pleasant and neutral images.

As anticipated, these results were somehow expected, in agreement with the hypothesis of hemispheric differences in emotional processing as dependent on stimulus valence. Nevertheless, the tDCS effects were contrary to the direction of most past findings, which showed an advantage of anodal stimulation in improving different behavioral measures (both in motor and in many non-motor tasks). We found that right anodal/left cathodal stimulation was associated to an enhanced recall of positive stimuli, which are supposed to be mainly processed by the left hemisphere, and we found an analogous configuration for left anodal/right cathodal stimulation and negative stimuli, supposed to be mostly analyzed by the right hemisphere. Rather than interpreting this pattern as being due to a hemisphere specialization for emotional stimuli contrary with respect to the one assumed by the specific-valence hypothesis (since we found reverse associations), we ascribed our reversed pattern of results to an opposite effect of tDCS on performance.

This unexpected finding helped us to reach the other, more methodological, aim of the present study, that is the investigation of the tDCS effects on emotional memory, in order to enrich the knowledge about this technique and on its capability of affecting cognitive processes and behavioral performance. In the present study, we found that a constant current of 1 mA, applied in fronto-temporal areas for 20 minutes, during the encoding phase of stimuli with different emotional content, did not seem to generally affect the following explicit memory test. Even better, given the double dissociation between the side of anodal stimulation and the kind of emotional stimuli better remembered (in the second analysis), data seem to be more in line with an interfering and detrimental effect of anodal stimulation on behavioral performance. In line with Boggio's argumentation on anodal stimulation effects on a different memory system [23], we suppose that this kind of stimulation, by increasing excitability, could induce interfering defocusing effects. In particular, anodal stimulation, being quite diffuse, could induce an enhanced activity in a large cortical network, which, through competition, could decrease the advantage of a more circumscribed network, naturally specialized to perform the target cognitive processes. In this regard, a variation of the present tDCS protocol, using a more anterior stimulation site (i.e., the dorsolateral prefrontal cortex) and not including temporal areas, could be useful in clarifying the mentioned defocusing effects of anodal stimulation. In addition, it has to be considered that, since we used a bilateral montage (in order to avoid confounds, and obtain a stimulation of equal spreading and intensity on the two hemispheres), it is difficult to establish the relative contribution of each kind of stimulation (i.e., anodal and cathodal) with regards to the effects we obtained. In fact, when we attribute an interfering effect to anodal stimulation, we need to keep in mind that such an effect can be mixed with a simultaneous and concurrent facilitatory effect of controlateral cathodal stimulation. In other words, in the present paradigm, behavioural performance can be influenced by a combination of cognitive interference exerted by anodal stimulation in one hemisphere and of cognitive facilitation exerted by cathodal stimulation in the controlateral hemisphere. Therefore, further studies are needed in order to evaluate the relative contribution of each kind of stimulation and of each hemisphere.

At the same time, we need to underline that these results could be potentially linked to many other factors, all related to specific tDCS parameters selected for the study. Indeed, we can not rule out the possibility that our pattern of findings could be strictly due to the site of stimulation (related to the orientation of the electric field), to its duration and/or intensity, and, finally, to the cognitive processes during the which the stimulation was applied (encoding phase, instead of, for instance, retention or retrieval phases). It is important to specify that when we assert that behavioral performance is influenced by stimulation applied during the encoding of emotional stimuli, we do not necessarily imply that the effects of such a stimulation start in (or are restricted to) this phase of memory processing. In fact, our data are also consistent with tDCS effects which start (or last) in the following phases of stimulus retention or retrieval. Further experiments that will selectively manipulating the phase of tDCS application will be conducted in order to specifically investigate this issue. Nonetheless, although the present paradigm cannot resolve this question, it should be considered as a first step toward a methodical exploration of emotional memory with tDCS.

Consequently, it is obvious that the systematic investigation of current stimulation parameters in any given cognitive domain is absolutely necessary in order to characterize optimal standard protocols. Indeed, although anodal stimulation has generally proven to be effective in ameliorating many cognitive functions and behavioral performances till now, in some circumstances, as in the present study, effects are not consistent with previous findings, possibly due to several factors, that need to be further analyzed. In these last cases different kinds of stimulation could hamper or selectively affect the processing of different kind of stimuli, with implications that must be considered in advance. Within the field of emotional memory, the selective influence of tDCS that we found with respect to stimuli with different affective valence is critical for its forensic and neurorehabilitative applications. The delineation of tDCS protocols suited to selectively enhance the memories of specific kinds of stimulus could indeed be fundamental to improve both eyewitness memory and the recovery of different kinds of patients (like amnesic or depressed people) from an impaired processing of emotional stimuli.
